# Drugs, alcohol and sexual health: opportunities to influence risk behaviour

**DOI:** 10.1186/1756-0500-1-27

**Published:** 2008-06-18

**Authors:** Robert Patton, Francis Keaney, Michael Brady

**Affiliations:** 1 National Addiction Centre, Institute of Psychiatry, Kings College London, UK; 2 Kings College Hospital NHS Foundation Trust, Denmark Hill, London, UK

## Abstract

**Background:**

Alcohol and drug consumption can affect judgment and may contribute towards an increased likelihood of engaging in risky sexual behaviour. In this cross sectional survey of clients attending STI services levels of drug and alcohol use were assessed using two standardised drug and alcohol screening instruments (the PAT and the SDS).

**Findings:**

The rates of hazardous alcohol consumption were similar to those found among patients attending A&E departments. Approximately 15% of clients indicated possible dependence on alcohol or other drugs, and these clients were likely to cite their substance use as related to their attendance, and to accept the offer of help or advice.

**Conclusion:**

The use of brief screening instruments as part of routine clinical practice is recommended. The STI clinic is well placed to identify substance use and to offer advice and/or onward referral to specialist services.

## Introduction

Research examining the prevalence of Sexually Transmitted Infections (STI) and risk behaviours amongst a population of drug users in treatment indicates a high level of unprotected sexual activity [1] (22%). Hwang et al [2] found that 62% of drug users in treatment had serological markers for at least one STI. Another study by Lally et al [3] indicated that 23% of women in short-term substance abuse treatment were infected. A review by Marx et al (1991) [4] found associations between drug use (particularly crack) and STIs.

High rates of hazardous drinking among clients were confirmed during a recent pilot study based in the sexual health clinic at St Mary's Hospital in inner-London [5], in this study 96 patients attending a walk-in clinic were screened, 27% were found to be drinking excessively and 13% thought their attendance in the clinic might be related to their consumption of alcohol. The relationship between drug taking and engagement in sexual activity (protected or unprotected) is important; however the establishment of a causal relationship between the two remains unproven [6]. STI services offer an opportunity to identify hazardous and harmful drug and alcohol users and to either offer them brief advice in the form of a leaflet, or refer them on to specialist services. This study examines the prevalence of substance use among clinic users, their perception as to the relationship between their substance use and clinic attendance and the extent to which they are willing to accept help and advice about their drug or alcohol usage.

## Method

Over a six week period, 700 consecutive clients attending an STI clinic of a busy South London hospital were approached by a junior doctor. At the end of the consultation, having obtained consent, the doctor administered the Brief Alcohol & Drug Screen; a composite measure that includes the Paddington Alcohol Test (PAT) [7] and Severity of Dependence Scale (SDS) [8,9]. Participants who indicated that they drank more than 8 (male) or 6 units (female) on a single occasion, or who admitted that their visit to the clinic was related to their drinking, were classified as hazardous drinkers. Those scoring 3+ on the SDS were regarded as possibly dependant. Participants identified as hazardous drug or alcohol users were offered a leaflet outlining how they could access help with their substance use problem, and asked if a referral were made to local drug/alcohol services whether they would attend.

## Results

Six hundred and fifty three clients consented to participate in the study (93%). The majority were male (59.4%). Participants were aged between 17 and 78, with most in the 25–44 age group (55.5%). The majority of clients drank alcohol (71%), with 28.2% identified as hazardous drinkers (39.5% of those who drank alcohol were hazardous drinkers). 13.8% of the sample smoked tobacco. Illicit substance use was not widespread among participants, with just 5.3% admitting cannabis use, and less than 0.5% other drugs. Almost a quarter of all hazardous alcohol users (and 14.7% of all participants overall) were SDS positive, indicating possible dependence. Table 1 indicates the proportion of Alcohol, Tobacco and Cannabis users who were SDS positive, who accepted the offer of help and who indicated willingness to attend a referral.

**Table 1:** Alcohol, tobacco and cannabis consumption

	**Prevalance** **% (N)**	**Attendance related** **% (N)**	**SDS+** **% (N)**	**Accepted help** **% (N)**	***Would attend *** ***% (N)***
**Alcohol (hazardous)**	28.0 (183)	18.6 (34)	24.6 (45)	14.2 (26)	*84.6 (22)*
**Cigarettes**	13.8 (90)	4.4 (4)	35.6 (32)	38.9 (35)	*91.4 (32)*
***Cannabis***	*5.3 (35)*	*8.6 (3)*	*54.3 (19)*	*40.0 (14)*	*100.0 (14)*

One in seven hazardous drinkers (14.2%) and half of those identified as SDS positive accepted an offer of help or advice, and of these 90% indicated that they would also accept a referral for an appointment with a specialist to discuss their alcohol or drug consumption.

Overall 6.6% of the sample thought that their attendance was related to their substance use. Binary logistic regression indicated that hazardous drinking did not predict the acceptance an offer of help/advice (OR 0.5, 95% CI 0.3 – 0.9), however SDS+ status did (OR 17.2, 95% CI 9.0 – 32.3). SDS+ status also predicted patients who indicated that their attendance was related to their substance use (OR 3.1, 95% CI 1.5 – 6.5).

## Discussion

Clinicians may be surprised at the low numbers of clients who related their attendance to substance use; however this might be expected, given that in other settings, such as Accident & Emergency departments, rates are similar [8].

The 2000 Psychiatric Morbidity Survey [11] reports hazardous alcohol consumption, tobacco and cannabis use in the UK general population to be 26%, 30% and 25% respectively. Participants in the study had similar rates of hazardous alcohol use, but much reduced rates of both tobacco and cannabis consumption. Interestingly, the rate of possible dependence to alcohol was six times the national rate (24.6% vs. 4%). These results suggest that harmful levels of alcohol consumption are associated with STI clinic attendance, and future research should attempt to determine if there is a causal relationship.

STI clinics are well placed both to identify substance use and to offer onward referral to those whose use is problematic. At present most STI services do not routinely screen for alcohol or other drugs. The identification and appropriate referral of those clients whose substance use may well be associated with their clinic attendance may not only help to reduce clinic workload, but also enable STI services to deliver an important public health message.

This study indicates that using a brief screening instrument for drugs and alcohol as part of routine clinical work is a feasible proposition. While clinicians may be reluctant to address issues of substance misuse in the clinic setting, many clients are certainly receptive to advice and onward referral, which we know will help reduce their consumption and may in turn reduce the likelihood of engaging in risk behaviours.

## Competing interests

The authors declare that they have no competing interests.

## Authors' contributions

RP, FK and MB designed the study and helped to draft the final manuscript. MB facilitated data collection. RP was responsible for data analysis. All authors read and approved the final manuscript.
